# Dietary Assessment of Older Korean Adults by Level of Plant Protein Intake

**DOI:** 10.3390/nu17121976

**Published:** 2025-06-11

**Authors:** Yong-Seok Kwon, Ye-Jun Kim, Jeong-Hun Song, Yangsuk Kim

**Affiliations:** 1 Department of Food Sciences, National Institute of Crop and Food Science, Rural Development Administration, Wanju 55365, Republic of Korea; selenium2012@korea.kr (Y.-S.K.); backyul99@korea.kr (Y.-J.K.); 2 Department of Agricultural Biology, National Institute of Agricultural Sciences, Rural Development Administration, Wanju 55365, Republic of Korea; jeonghuns@korea.kr

**Keywords:** plant protein, Korean older adult, Korea national health and nutrition examination survey, dietary quality, food intake

## Abstract

Background/Objectives: The purpose of this study is to evaluate the dietary habits of older Korean adults according to their level of plant protein intake. Methods: To conduct this study, the daily plant protein intake of 4254 older adults aged 65 years and older who participated in the dietary survey (24-h recall method) of the 2016~2018 Korea National Health and Nutrition Examination Survey (KNHANES) was divided into quintiles. And the association among plant protein quintiles and general characteristics, health-related behaviors, dietary habits, nutrient intakes, and Korean Healthy Eating Index (KHEI) was examined. Results: For the energy contribution of protein, more than 90% of all groups from Q1 through Q5 were in the Acceptable Macronutrient Distribution Range (AMDR, 7–20%) for protein. In the case of food security, the proportion tended to increase by about 11 percentage points from Q1 (43.4%) to Q5 (54.4%) according to plant protein quintiles, and while food insecurity was above 50% from Q1 to Q3, the percentage tended to decrease as the plant protein quintile increased from Q4 (48.3%) to Q5 (45.3%). Overall, the low plant protein intake group, especially the Q1 to Q3 group, was found to have an inadequate intake of nutrients, including total protein, compared to the recommended intake. In addition, among the components of the KHEI score, the score for the item “consumption of multigrain rice” was found to be very low. Conclusions: Based on these findings, dietary education or guidelines are needed to increase individual awareness, as well as the development of dietary content at government level to support this.

## 1. Introduction

Proteins serve a nutritional function, providing amino acids and nitrogen compounds necessary for the normal growth and maintenance of the human body, while also serving as a source of energy, producing 4 kcal per gram [1]. It is involved in normal growth, physiological functions, and life support as a component of body tissues such as bones, muscles, skin, nails, and hair, as well as antibodies, hormones, and enzymes. It is also one of the three major nutrients, along with carbohydrate and fat, that provide the body with the energy it needs [1,2].

According to the National Health Statistics published by the Korea Disease Control and Prevention Agency, the protein intake of adults aged 65 years and older in South Korea in 2021 was 56.5 g, and the ratio of protein intake to the Recommended Nutrient Intake (RNI) was 113.9%, with an energy contribution of 14.6% [3]. Considering that the AMDR of protein in the Dietary Reference Intakes for Koreans (KDRIs) is 7–20% [1], the protein intake level of older Korean adults seems to be adequate overall, but there are still concerns about inadequate intake, as shown in a previous study [2]. A study assessing protein intake among older adults aged 60 years and older using data from the 2013–2014 KNHANES reported that 30.8% of males and 42.6% of females consumed less than the Estimated Average Requirement (EAR) for protein per unit body weight [4]. A study analyzing adults aged 65 years and younger who participated in the 2016~2018 KNHANES found that 66.5% of males and 58.2% of females met the RNI of 0.91 g/kg/day of protein per unit body weight [5]. Therefore, it is important to consider not only the absolute amount of protein consumed, but also its relationship to other indicators of protein intake in order to accurately assess protein intake.

On the other hand, animal protein is considered high-quality protein due to its high content of all essential amino acids and high bioavailability [6]. However, animal protein intake tends to decrease with age for various reasons such as loss of appetite, financial constraints, and poor digestive ability [7]. In particular, in the case of older adults, plant protein intake was reported to account for a larger proportion of total protein consumption than animal protein intake [8]. Among these plant proteins, legume, nut, and seed (LNS) proteins refer to a category of plant proteins that is rich in essential amino acids and is considered high-quality protein. It has been reported that these LNS proteins can be an important source of protein for people who do not consume sufficient amounts of animal protein [9]. Previous studies have reported that plant proteins can have a positive effect on muscle mass and strength [10,11,12,13,14].

The association between protein intake and diabetes risk has been investigated in several large cohort studies. The EPIC-InterAct study included 12,403 cases of type 2 diabetes from eight European countries and a subcohort of 16,154 adults who were followed for an average of 12 years. For every 10 g increase in animal protein intake, the risk of diabetes increased by 5%, with a stronger association in women. In addition, plant protein intake was not significantly associated with diabetes risk [15]. In the Zutphen Elderly Study, which followed 272 men aged 65–84 years for 15 years, higher plant protein intake was associated with lower systolic (−2.9 mmHg) and diastolic (−1.7 mmHg) blood pressure after 5 years. In this cohort of older men, no significant association was observed between animal protein intake and blood pressure [16]. An intervention study in 15 postmenopausal women (mean age 61 years) with abdominal obesity compared two high-protein diets (22% of energy from protein) for 4 weeks. When participants consumed 30 g/day of soy protein instead of meat protein, total cholesterol was reduced by 4% and LDL cholesterol was reduced by 9% compared with the mixed protein diet. The soy protein diet was also reported to improve insulin sensitivity [17]. A cross-sectional analysis of 8693 Chinese adults aged 18–75 years found that those who consumed more protein from refined grains and red meat had a higher risk of diabetes, whereas those who consumed more protein from legumes and seafood had a lower risk of diabetes. This previous study reported that the association between protein intake and diabetes may vary depending on the source of protein [18]. Summarizing these results [15,16,17,18], several studies have reported the association between chronic diseases such as diabetes and cardiovascular disease and intake of animal and plant protein foods, but most of them have observational study designs or the number of study subjects is small, so interpretation is limited.

Therefore, this study aims to utilize data from the KNHANES to determine the current status of plant protein intake among the older Korean adults. We also examine the dietary habits and dietary patterns associated with plant protein intake among the older Korean adults. Based on these results, we hope to contribute to the development of sustainable dietary guidelines for the older adults. This will serve as an important basis for developing policies and programs to improve the dietary status of the older adults in the future.

## 2. Materials and Methods

### 2.1. Research Data and Subjects

This cross-sectional study used KNHANES data from 2016 to 2018. KNHANES is a continuous surveillance system that assesses the nutritional health status and prevalence of chronic diseases in a large, representative sample of the Korean population. KNHANES is conducted annually by the Korea Disease Control and Prevention Agency and consists of a health interview, a health examination, and a dietary survey [19,20].

The main research subjects were selected based on several previous studies [20,21,22,23], and the procedure was as follows. This study used raw data (*n* = 30,551) from the 2016–2018 KNHANES. The main study population was older adults aged 65 years and older, who participated in the health and diet survey (24-h recall survey) (*n* = 4956). Based on the methods used in previous studies [22,23], we excluded from the analysis those with a total daily energy intake of less than 500 calories or more than 5000 calories (*n* = 72) and those with missing data from the dietary survey (*n* = 630). As a result, 4254 subjects were finally selected. The Korea Disease Control and Prevention Agency (KDCA) Institutional Review Board approved the KNHANES data used in this study (IRB approval numbers: 2018-01-03-P-A). Among these, the 2016 and 2017 KNHANES were exempt from review regarding research ethics based on the Bioethics and Safety Act from 2015 to 2017 [24].

### 2.2. Classification of Protein Intake

A trained interviewer in each participant’s home using the 24-h dietary recall method assessed dietary intake. Nutrient content data for each food were linked to the Korean Food Composition Table published by the Rural Development Administration [25] to estimate total protein, animal protein, and plant protein. According to the KNHANES food group classification, total daily plant protein intake includes proteins found in “cereals or grains”, “potatoes or starches”, “legumes and their processed products”, “seeds and nuts”, “vegetables”, “mushrooms”, “fruits”, “seaweeds”, or other plant foods and products made from these foods. Total daily animal protein intake was calculated from “meat”, “fish”, “eggs”, “milk or its processed products”, and all products made from other animal foods. Other protein included protein from miscellaneous foods, including baked goods and alcoholic beverages, based on classifications from previous study [26].

### 2.3. General Characteristics

The analysis was conducted with reference to the survey subjects’ gender, age, marital status, education level, region, employment status, and household income. Age was classified into two groups (65–74 years old and >75 years old), marital status was classified as single or married, and education level was classified into three categories (less than a high school diploma, a high school diploma, or college degree or higher). The region field was classified into city or rural area using dong/eup/myeon, and employment status was divided into employed or unemployed. Household income was divided into upper, middle–upper, middle–lower, and lower.

### 2.4. Health Behavior

Questions related to health behavior were smoking status, alcohol consumption level, stress level, exercise frequency, and weight status. Smoking status was divided into “non-smoker”, “former smoker”, and “current smoker”; alcohol consumption level was classified into four categories based on the frequency of drinking (4 or more times per week, 2–3 times per week, 1–4 times per month, or less than 1 time per month). The stress level was divided into four categories (“severe stress”, “moderate stress”, “mild stress”, or “no stress”). Four categories were assigned for exercise frequency (less than 1 day/week, 1–2 days/week, 3–4 days/week, or more than 5 days/week). Weight status was classified according to the standards of the World Health Organization’s Asia-Pacific Region and the Korean Society for the Study of Obesity [27,28]. Also, weight status was classified using the BMI (kg/m^2^) index; a BMI of <18.5 kg/m^2^ was classified as underweight, 18.5–23.0 kg/m^2^ as normal, 23.0–25.0 kg/m^2^ as overweight, and ≥25.0 kg/m^2^ as obese. KNHANES data include measurements of the subjects’ handgrip strength to determine their muscle strength distribution. A digital dynamometer (TAKEI, Niigata, Japan) was used to measure hand force. Each hand was measured three times, alternately, leading to a total of six times. In this study, as in the previous one, the maximum value of the dominant hand was used as the final force value. We evaluated the decrease in muscle strength using the 2019 Asian Working Group for Sarcopenia (AWGS) criteria [29,30]. We defined a handgrip strength less than baseline (male: 28.0 kg, female: 18 kg) as the low handgrip strength (LHS) group, and others as the normal or higher handgrip strength (NHS) group.

### 2.5. Dietary Behavior

Five questions investigated dietary behaviors: frequency of breakfast, snack, location of meals served, frequency of eating out, and food security. Breakfast frequency was classified as 5–7 times per week, 3–4 times per week, 1–2 times per week, or rarely. Snack consumption answers were either yes or no. For the location of meals served, home, commercial locations, and institutional locations were each classified as yes or no. Eating-out frequency was divided into six categories: more than 1 time/day, 5–6 times/week, 3–4 times/week, 1–2 times/week, 1–3 times/month, and rarely. Food insecurity has been included in the dietary questionnaire since the 2005 KNHANES, and the question “Which of the following best describes your family’s diet in the past year?” was classified into the “enough food secure” category of “everyone in our family could eat enough food and a variety of foods as much as they wanted” and the “mildly food insecure” category of “everyone in our family could eat enough food but couldn’t eat different kinds of foods” based on previous studies [31,32]. The “moderately/severely food insecure” category of “there was sometimes or often not enough food because it was economically difficult” was also classified and used in this study.

### 2.6. Food and Nutrient Intake

The dietary intake survey in the KNHANES was conducted using a 24-h recall method in which all food intake contents were for one day before the survey was answered. Food intake was categorized into 17 food groups (cereals and grain products, potatoes and starches, sugars and sweets, legumes and their products, seeds and nuts, vegetables, mushrooms, fruits, meat, poultry and its products, eggs, fish and shellfish, seaweed, milk and dairy products, oils and fats, beverages, seasonings, and other food). Additionally, energy, macronutrient (carbohydrate, protein, and fat), and micronutrient (calcium, phosphorus, iron, sodium, potassium, vitamin A, thiamine, riboflavin, niacin, and vitamin C) intake were analyzed, and the energy distribution ratio of macronutrients (carbohydrate, protein, and fat) was calculated.

### 2.7. Korean Healthy Eating Index (KHEI)

The KHEI is an indicator developed by the KDCA to assess adherence to the national dietary guidelines and to comprehensively evaluate the dietary lifestyle and quality of Koreans [33,34]. The KHEI has 14 components (Table 1), eight of which assess the adequacy of recommended food and nutrient intakes (breakfast intake and consumption of multiple grains, total fruits, fresh fruits, total vegetables, vegetables excluding kimchi and pickles, meat, fish, eggs and legumes, milk and dairy products), three evaluate the intake of foods and nutrients for which restricted consumption is recommended (percentage of energy intake from sweets and beverages, saturated fatty acids, and sodium), and three evaluate the balance of energy intake (adequate energy intake, and percentage of energy intake from carbohydrates and fats). The maximum possible score on the KHEI is 100 points. Some of the components had a weight of 5 points each (multi-grains, fruits, vegetables, percentage of energy intake from carbohydrates and fats, adequate energy intake); the rest had weights of 10 points, given their importance. The KHEI score was outlined in the raw KNHANES data.

### 2.8. Statistical Analysis

The KNHANES data were collected using stratified multistage sampling rather than simple random sampling. So, the collected data were considered for statistical analysis when applying the weight variable (variable name: Wt_ntr), stratification variable (variable name: KSTRATA), and cluster variable (variable name: PSU, Primary Sampling Unit) to apply the stratification and multistage sampling design according to the statistical analysis procedure contained in the guideline for KNHANES [35]. All statistical analyses were conducted using the survey procedure of the SAS ver.9.4 package (SAS Institute, Cary, NC, USA), and the significance was set at *p* < 0.05. Among them, for categorical variables such as general information, dietary factors, and health-related factors, frequency (*n*) and weight percentage (weight %) were obtained through frequency analysis (using the surveyfreq procedure of SAS), and Rao-Scott chi-square test (using the surveyfreq procedure of SAS) was performed for significance test. For the quantitative variables, a descriptive analysis (surveymean procedure of SAS) was performed to present the mean and standard error. To test the significance of these continuous variables, linear regression analysis was performed using the surveyreg procedure of SAS to obtain *p* for trend values. For nutrient and food intakes and the KHEI score, linear regression analysis (using the surveyreg procedure of SAS) was performed to obtain *p* for trend values adjusted for gender, age, and energy intake. In addition, comparisons of handgrip strength, nutrient and food intakes, and KHEI score according to plant protein intake quintile were tested for significance using ANCOVA (using surveyreg procedure of SAS), adjusted for age, gender, and energy intake, and Tukey’s multiple range comparison test was used when there were significant differences between groups.

## 3. Results

### 3.1. Median, Mean, and Range of Plant Protein Intake by Quintile of Plant Protein of Older Korean Adults

Table 2 shows the results for median, mean, and range of plant protein intake by quintile of plant protein of older Korean adults. Median of plant protein intake was 17.2 g (mean: 16.2 g, intake range: 0–21.0 g) for the Q1 group, 23.9 g (mean: 24.1 g, intake range: 21.01–27.25 g) for the Q2 group, 30.5 g (mean: 30.5 g, intake range: 27.26–34.11 g) for the Q3 group, and 38.7 g (mean: 38.8 g, intake range: 34.12–44.36 g) for the Q4 group, and the Q5 group showed 53.4 g (mean: 57.7 g, intake range: 44.37–157.56 g).

### 3.2. General Characteristics

General characteristics are as shown in Table 3. There was a significant difference in gender, age, education, occupation, and household income (*p* < 0.001). However, there was no significant difference in general characteristics between marital status and region among quintile groups of plant protein.

### 3.3. Health-Related Behaviors

Health-related behaviors are shown in Table 4. There was a significant difference in smoking status, drinking status, stress level, exercise status, dietary supplement use, and chewing ability (*p* < 0.05). Among these, the exercise variable’s item of exercising more than five times a week tended to increase by approximately 9.0% points as plant protein intake increased from Q1 (4.1%) to Q5 (13.6%). Very inconvenient for chewing ability tended to decrease by less than half as the plant protein quintile increased from Q1 (15.7%) to Q5 (7.4%). 

### 3.4. Measuring Handgrip Strength and Assessing Sarcopenia

The results of measuring handgrip strength and assessing sarcopenia were as follows (Table 5). In the LHS group whose handgrip strength was below the baseline (male: 28.0 kg, female: 18.0 kg), the proportion of participants decreased by approximately 23.0 percentage points as plant protein intake increased from the Q1 to the Q5 (*p* < 0.001). Furthermore, the mean handgrip strength of the subjects tended to increase significantly by about 7.4 kg in Q5 (26.6 kg) compared to Q1 (19.2 kg) (unadjusted *p* for trend < 0.001). However, after adjusting for gender, age, and energy intake, there was no significant difference in grip strength between the Q1 and Q5 groups (adjusted p for trend=0.0789). 

### 3.5. Dietary Behaviors

Dietary behaviors are shown in Table 6. All variables except the institution location variable had significant differences (*p* < 0.001). Among these, breakfast 5–7 times/week tended to increase by approximately 8.1% points as plant protein intake increased from Q1 (87.0%) to Q5 (95.1%) groups. In addition, the percentage of subjects who ate at each meal prepared location is as follows. The incidence of meals provided at commercial locations tended to increase by about 11.3% points from Q1 (65.2%) to Q5 (76.5%). The incidence of meals provided at home tended to increase by about 12.3% points from Q1 (83.3%) to Q5 (95.7%). For the eating-out frequency variable, the frequency of eating out more than five times a week increased from Q1 to Q5. Conversely, the category of eating out rarely (less than once a month) tended to decrease by about 20.0% points from Q1 (34.6%) to Q5 (14.9%). Lastly, the results of food security are as follows. The enough food secure item tended to increase by approximately 11.0% points from Q1 (43.4%) to Q5 (54.4%), while the mild food insecure and moderate/severe food insecure items tended to decrease from Q1 to Q5.

### 3.6. Percentage Contribution of Food Groups to Total Protein Intake by Quintile of Plant Protein

The results for percentage contribution of food groups to total protein intake by quintile of plant protein are shown in Table 7. First, the intake flavor for each food group is as follows. Consumption of “total food” and plant food groups (“cereals and grains”, “potatoes and starches”, “vegetables”, and “seaweeds”) tended to increase significantly (unadjusted *p* for trend < 0.001, adjusted *p* for trend < 0.05). Intake of animal food sources such as “meat and poultry”, “eggs”, “fish and shellfish”, and “milk and dairy products” increased significantly before adjusting for gender, age, and energy intake (unadjusted *p*-value for trend <0.001, “milk and dairy products” were not significant before adjusting.), and showed a decreasing trend after adjustment for gender, age, and energy intake (adjusted *p*-value for trend <0.01). The oils and fats “seasonings” and “other food” food groups, which contain both animal and plant foods, showed a similar trend to the animal food group.

When looking at protein intake by food group, there was a significant increase in plant-based food groups, including “protein intake from whole foods”, “cereals and grains”, “legumes”, “seeds and nuts”, “vegetables”, “mushrooms”, and “seaweed (unadjusted *p* for trend < 0.001, adjusted *p* for trend < 0.05). Consumption of animal food groups such as “meat and poultry”, “eggs”, “fishes and shell fishes”, and “milks and dairy products” tended to increase significantly before adjusting for gender, age, and energy intake variables (unadjusted *p* for trend < 0.001). However, after adjusting gender, age, and energy intake variables, there was a tendency to decrease (adjusted *p* for trend < 0.05). “Seasonings” and “beverages”, which are a mixture of animal and plant foods, showed an increasing trend from Q1 to Q5 like the plant food group (unadjusted *p* for trend < 0.001, adjusted *p* for trend < 0.05).

When analyzing the contribution of each food group to total protein food intake, “cereals and grains”, “beans”, “mushrooms”, “vegetables”, and “seeds and nuts” showed a significant percentage increase from Q1 to Q5 (unadjusted *p* for trend < 0.05, adjusted *p* for trend < 0.01, “seaweeds”, and “cereals and grains” had no significant difference before adjusting.). On the other hand, animal food groups such as “meat and poultry”, “eggs”, “fishes and shell fishes”, and “milks and dairy products” showed a decreasing trend (adjusted *p* for trend < 0.001, “eggs”, “fishes and shell fishes” had no significant difference before adjusting.). “Seasonings”, which is a mixture of animal and plant foods, showed a tendency to decrease like the animal food group, and “beverages” showed a tendency to increase like the plant food group (unadjusted *p* for trend < 0.001, adjusted *p* for trend < 0.001).

### 3.7. Plant, Animal, and Other Protein Intake of Older Korean Adults by Quintile of Plant Protein

**Figure 1:**
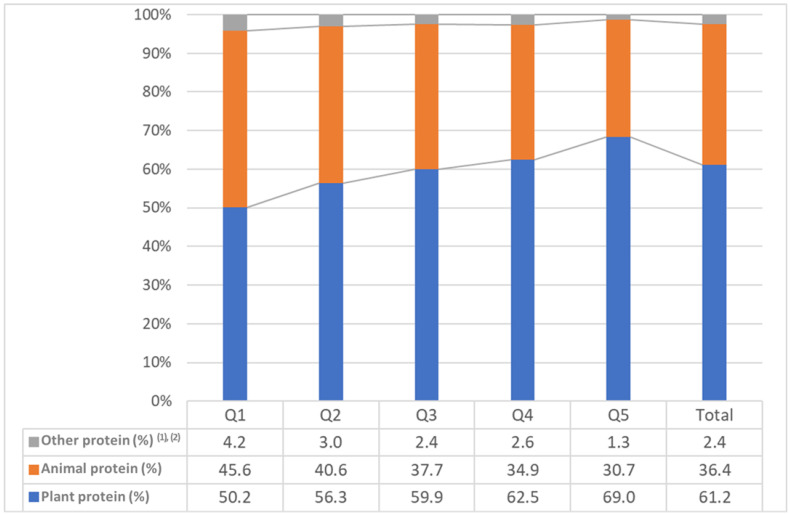
Contribution of plant, animal, and other proteins to total protein intake by plant protein quintiles of older Korean adults. ^(1)^ other protein included miscellaneous foods, baked goods, and alcoholic beverages; ^(2)^ weighted %.

Figure 1 shows the contribution of plant, animal, and other proteins to total protein intake by plant protein quintiles of older Korean adults. The contribution of plant protein to total protein was found to be between 50 and 69%. Conversely, the contribution of animal protein to total protein was found to decrease from Q1 (45.6%) to Q5 (30.7%).

### 3.8. Nutrient Intake According to Plant Protein Quintile

Nutrient intakes by plant protein quintile are shown in Table 8. Intakes of vitamin A, carotene, riboflavin, vitamin C, and niacin tended to increase from Q1 to Q5 before adjusting for gender, age, and energy intake (unadjusted *p* for trend <0.001). However, there was no significant difference after adjustment. Retinol and vitamin D, on the other hand, showed a significant increase before adjustment but a trend toward decrease after adjustment (unadjusted *p* for trend <0.01, adjusted *p* for trend <0.01). The remaining nutrients and energy contributions from protein, fat, and carbohydrates tended to increase with or without adjustment (unadjusted *p* for trend <0.05, adjusted *p* for trend < 0.05).

### 3.9. Total, Animal, and Other Protein Intake of Older Korean Adults by Quintile of Plant Protein

Table 9 shows the results for energy contribution of protein older Korean adults by quintile of plant protein. In the AMDR of protein according to plant protein quintile, the appropriate group (7–20%) was found to be more than 90% from Q1 to Q5.

### 3.10. KHEI Total and Component Scores of Older Korean Adults by Quintile of Plant Protein Intake

The results for KHEI total and component scores of older Korean adults by quintile of plant protein intake are shown in Table 10. Because of calculating the KHEI, the KHEI for all older adults was 67.4 points. The Q1 group scored 60.0 points, the Q4 group scored the highest at 70.9 points, and the Q5 group scored 70.7 points. Looking at each item of the KHEI for all subjects, “percentage of energy from sweets and beverages” had the highest score of 9.5 out of 10, followed by “have breakfast” with 9.5 points and “percentage of energy from sweets and beverages” with 9.5 points out of 10. “Saturated fatty acid” was high at 9.1 points, “sodium intake” was high at 7.9 points, and “meat, fish, eggs, and beans intake” was high at 6.6 points. On the other hand, “percentage of energy from carbohydrate” was the lowest at 1.7 points out of 5, and “multiple grain intake”, “total fruit intake”, and “raw fruit intake” were also less than half of the total score. When comparing the scores of the component items by quintile of plant protein intake, the Q1 and Q2 groups, which had the lowest total score, had a very low score for the “multigrain rice intake” item compared to the other groups. Overall, item scores for “total fruit intake” and “fresh fruit intake” showed a significant increase from Q1 to Q5 (unadjusted *p* for trend *p* < 0.001), but not when adjusted for gender, age, and energy intake. Item scores for “milk and dairy intake”, “sodium intake”, “percent energy from carbohydrates”, and “percent energy from fat” tended to decrease from Q1 to Q5 when adjusted for gender, age, and energy intake (adjusted *p* for trend < 0.05).

## 4. Discussion

This study aimed to assess the dietary habits of older Korean adults by level of plant protein intake using data from the KNHANES. To conduct this study, we divided plant protein intake into quintiles and examined the association between general characteristics and dietary habits of food/nutrient intake and KHEI.

This present study found that older Korean adults most commonly consumed plant protein from “cereals and grain products” (31–35%), regardless of age group. From Q1 to Q5, the contribution of “cereals and grain products” increased by about 3.2 percentage points, while the contribution of protein from legumes, nuts, and seeds (LNS) increased by more than 11 percentage points (Q1: 4.3%, Q5: 15.5%). These LNS proteins are a source of essential amino acids, including leucine, lysine, and valine [14,36,37]. Previous studies have shown that leucine regulates protein synthesis in skeletal muscle. Also, leucine stimulates human muscle protein synthesis through several pathways [14,38]. A meta-analysis found that soy supplementation resulted in gains in strength and muscle mass similar to whey protein [39]. A study using probabilistic approaches and dietary modeling of 1678 adults using data from the French National Dietary Survey reported that LNS proteins can replace animal proteins more effectively than other plant proteins and maintain protein and amino acid adequacy [40]. In general, however, plant proteins are known to be incomplete proteins, missing one or more essential amino acid [9,14]. A literature review of dietary proteins and amino acids in vegetarian diets reported that among plant proteins, LNS proteins are high-quality plant proteins that are rich in essential amino acids. Additionally, LNS proteins have also been reported to be an important source of protein for vegetarians and those who do not consume enough animal protein [9]. Another previous study analyzed the association between plant protein intake and sarcopenia in Koreans aged 50+ and found higher intake of total plant protein and LNS protein linked to a lower prevalence of sarcopenia [14]. In this present study, the LHS group (sarcopenic group with maximum handgrip strength below baseline) showed a trend of approx. 23 percent point decrease in plant protein intake from Q1 (45.8%) to Q5 (22.7%). The subjects’ grip strength increased by about 7.4 kg from Q1 (19.2 kg) to Q5 (26.6 kg). This is similar to the results of previous studies related to total plant protein intake and the results of this present study. However, after adjusting for gender, age, and energy intake, there was no significant difference in grip strength between the Q1 and Q5 groups. To explain these results more specifically, looking at the general characteristics of this present study, we found that increasing plant protein intake from Q1 to Q5 was associated with decreasing age, increasing male proportion, and increasing energy intake. We believe that the effects of these variables on hand grip strength were offset after adjustment. We recommend that future studies use relative plant protein intake (the energy contribution of plant protein) rather than absolute plant protein intake as a baseline and break down the analysis by general characteristics such as gender and age. Additionally, clinical intervention studies are needed to confirm the association between plant protein intake and various diseases, but the results of this present study and previous studies included in this discussion suggest that a diet containing plant proteins with adequate LNS protein may be helpful in maintaining muscle health in older adults.

In this present study, calcium intake in the Q1–Q4 groups ranged from about 100 to 330 mg, which is lower than the EAR of 600 mg for KDRIs [1]. Previous studies have reported that the majority of older adults aged 65 years and older in the 2013–2017 KNHANES did not reach the EAR for calcium intake from food. In the 65–74 age group, 70.6% of men and 72.2% of women were below the EAR, and in the 75+ age group, 79.3% of men and 96.6% of women were below the EAR [1,41,42]. The results of this present study suggest that calcium intake among older Korean adults is lower than the recommended intake, regardless of the level of plant protein intake. Previous studies on persistent calcium deficiency in Korean adults aged 20 years and older also highlight the urgent need to increase the availability of calcium-rich foods and implement targeted interventions to increase calcium intake among those most affected by calcium deficiency, especially older adults and female [43]. When we synthesize the results of this present study with the results of previous studies, we believe that we should seek measures to increase calcium intake among Koreans, including the older adults, through cooperation between academia, research institutes, and government agencies. Meanwhile, for sodium, the 2020 KDRIs proposed a Chronic Disease Risk Reduction Intake (CDRRI) of 2100 mg for those aged 65 to 74 years and 1700 mg for those aged 75 years and older [1]. In this present study, older adults in groups Q2 to Q5 all had sodium intakes that exceeded the CDRRI, suggesting a need for caution. Meanwhile, vitamin A intake in this present study tended to be below the EAR in older men from Q1 to Q5, while older women were about 36 µg RAE above the EAR of 410 µg RAE from Q5. Finally, vitamin C is an essential nutrient for the normal physiological functioning of the human body. Therefore, it has been reported that inadequate vitamin C intake can lead to anemia, infection, capillary bleeding, delayed wound healing, muscle degeneration, and neurological disorders, including scurvy, which is the most common disease [44]. In this present study, the vitamin C intake of the older adults was analyzed, and all of the groups from Q1 to Q4, except for Q5 (80.78 mg), were below the EAR of 75 mg, except for Q5, suggesting that measures should be taken to improve this situation. As for the energy contribution of carbohydrates, all older adults in the Q1 to Q5 groups were about 6–10% above the recommended percentage of 55–65% as suggested by the KDRIs [1], which may require attention. According to previous studies, more than half of Korean adults diagnosed with diabetes, hypertension, and metabolic syndrome consume more than 70% of their total energy from carbohydrates, and this trend was reported to be more pronounced in the 60+ age group [45]. Considering the results of previous studies and this present study, it is believed that measures should be taken to improve dietary habits at the national level for older adults in groups from Q1 to Q3, which are groups with low plant protein intake. In particular, when looking at the food insecurity of these subjects, it was over half of 50–56%, and since energy and nutrient intake is generally insufficient; efforts are needed to improve their dietary habits. The pattern of change in retinol and vitamin D intake revealed a complex relationship between plant protein intake and nutrient intake. Unadjusted results showed that retinol and vitamin D intake increased significantly with increasing plant protein intake, but after adjusting for gender, age, and energy intake, consumption of these nutrients tended to decrease. While these results need further confirmation, factors such as gender, age, and energy intake may have a significant impact on nutrient intake patterns. In addition, in older adults, increasing plant protein intake may decrease intake of other important nutrients, suggesting that nutritional status in older adults may be influenced by multiple factors and that simply increasing intake of a particular nutrient may not be sufficient. Future studies, such as long-term follow-up cohorts or clinical interventions, are needed to further investigate the effects of increased plant protein intake on overall dietary patterns and nutrient adequacy in older adults.

According to the results of this present study, plant protein accounted for 50~68% of total protein intake. According to a report published by the Food and Agriculture Organization of the United Nations (FAO), plant protein accounts for a higher proportion of total protein intake than animal protein in older adults [8]. In previous study by Lee and Shin, which analyzed the protein intake trends of adults aged 19 years and older in Korea by analyzing KNHANES data from 1998 to 2018, 63% of total protein intake was plant protein and 37% was animal protein in 1998, but the gap narrowed to 57% and 43% for animal protein in 2016–2018, and the proportion of plant protein still exceeded half. For this reason, the previous study reported that “cereals and processed cereal products (23.9%)” contributed greatly to plant protein intake [46]. Another study reported that rice, a staple food, was the food that contributed most to protein intake in Koreans [47]. In addition, the result of this present study confirmed that the food group that contributed the most to plant protein intake was “cereals and grain products (31–35%)”. Considering these previous studies and the results of this present study, it is thought that plant protein intake, especially grain protein intake, is an important part of the diet of older Korean adults.

There was a significant result between breakfast frequency and plant protein intake. The proportion of those who ate breakfast 5–7 times a week tended to increase by about 8.1%p from Q1 (87.0%), which had the lowest plant protein intake, compared to Q5 (95.1%), which had the highest intake. It is thought that regular breakfast can be seen as an indicator of healthy lifestyle habits. Therefore, it is thought that the group with a high intake of plant protein is likely to have healthier lifestyle habits overall. In addition, it is believed that the importance of breakfast should be emphasized when developing policies or dietary programs to improve the nutritional status of the older adults.

Overall, the low plant protein intake group, especially the Q1 to Q3 group, was found to have an inadequate intake of nutrients, including total protein, compared to the recommended intake, and food insecurity was more than 50%. In addition, among the components of the total KHEI score, the score for the item “consumption of multigrain rice” was found to be very low. This is thought to be due to the high intake of refined grains such as white rice in the cereal group of carbohydrates. In order to improve these eating habits, dietary education or guidelines are needed to increase individual awareness, as well as the development of dietary content at government level to support this.

This study has several limitations. It is a cross-sectional study using the KNHANES, and like the previous study, it is not a study of the same subjects, so it could not examine changes in individual energy, nutrient, and food group intakes as a long-term follow-up cohort study [25,48]. In addition, the dietary survey of the KNHANES is conducted by 24-h recall method of meals consumed the day before the survey, so there are limitations in reflecting the subjects’ daily intake [48]. Despite these limitations, this study is considered meaningful because it used KNHANES, which is a representative dietary data source in Korea, and secured a representative sample.

## 5. Conclusions

This study aimed to assess the dietary habits of older Korean adults by level of plant protein intake using data from the KNHANES. The results of this study were as follows. For the energy contribution of protein, more than 90% of all groups from Q1 through Q5 were in the AMDR (7–20%) for protein. In the case of food security, the proportion tended to increase by about 11 percentage points from Q1 (43.4%) to Q5 (54.4%) in the covered group, and food insecurity was over 50% from Q1 to Q3, but tended to decrease as the plant protein quintile increased. Overall, the low plant protein intake group, especially the Q1 to Q3 group, was found to have an inadequate intake of nutrients, including total protein, compared to the recommended intake, and food insecurity was more than 50%. In addition, among the components of the KHEI score, the score for the item “consumption of multigrain rice” was found to be very low. This is thought to be due to the high intake of refined grains such as white rice in the cereal group of carbohydrates. In order to improve these dietary habits, dietary education or guidelines are needed to increase individual awareness, as well as the development of dietary content at government level to support this.

## Figures and Tables

**Table 1:** Korean Healthy Eating Index components and standards for scoring.

Classification (No. of Components)	Components	Score Range	Standard for Maximum Score	Standard for Minimum Score
Adequacy (8)	Have breakfast	0–10	5–7 times/week	0 times/w
	Mixed grains intake	0–5	≥0.3 serving/day	0 serving/d
	Total fruits intake	0–5	Men aged 65 years and overs: ≥2 serving/day Women aged 65 years and overs: ≥1 serving/day	0 serving/d
	Fresh fruits intake	0–5	Men aged 65 years and overs: ≥1 serving/day Women aged 65 years and over: ≥0.5 serving/day	0 serving/d
	Total vegetables intake	0–5	Men aged 65 years and overs: ≥8 serving/day Women aged 65 years and overs: ≥6 serving/day	0 serving/d
	Vegetables intake excluding Kimchi and pickled vegetables intake	0–5	Men aged 65 years and overs: ≥5 serving/day Women aged 65 years and overs: ≥3 serving/day	0 serving/d
	Meat, fish, eggs, and beans intake	0–10	Men aged 65 years and overs: ≥4 serving/day Women aged 65 years and overs: ≥2.5 serving/day	0 serving/d
	Milk and milk products intake	0–10	≥1 serving/day	0 serving/d
Moderation (3)	Percentage of energy from saturated fatty acid	0–10	≤7% of total energy intake	>10% of total energy intake
	Sodium intake	0–10	≤2000 mg/day	>6500 mg/d
	Percentage of energy from sweets and beverages	0–10	≤10% of total energy intake	>20% of total energy intake
Balance of energy intake (3)	Percentage of energy from carbohydrate	0–5	55–65% of total energy intake	<50% or >75% of total energy intake
	Percentage of energy intake from fat	0–5	15–30% of total energy intake	<10% or >35% of total energy intake
	Energy intake	0–5	75–125% of the estimated energy intake requirement (EER)	<60% or >140% of EER

**Table 2:** Median, mean, and range of plant protein intake by quintile of plant protein.

	Q1 (*n* = 850)	Q2 (*n* = 851)	Q3 (*n* = 851)	Q4 (*n* = 851)	Q5 (*n* = 851)	Total (*n* = 4254)
Intake range (min-max)	0–21.00	21.01–27.25	27.26–34.11	34.12–44.36	44.37–157.56	0–157.56
Median (g/day)	17.2	23.9	30.5	38.7	53.4	30.2
Mean (g/day)	16.2 (15.91–16.53) ^(1)^	24.1 (23.90–24.22)	30.5 (30.38–30.66)	38.8 (38.62–39.05)	57.7 (56.63–58.78)	33.2 (32.52–33.87)

^(1)^ Mean (95% confidence limits).

**Table 3:** General characteristics of older Korean adults by quintile of plant protein intake.

Variables	Q1 (*n* = 850)		Q2 (*n* = 851)		Q3 (*n* = 851)		Q4 (*n* = 851)		Q5 (*n* = 851)		Total (*n* = 4254)		*p*-Value ^(1)^
	*n*	% ^(2)^	*n*	%	*n*	%	*n*	%	*n*	%	*n*	%	
Total	850	20.5	851	20.0	851	20.2	851	20.0	851	19.3	4254	100.0	-
Gender													<0.0001
Male	220	25.1	291	32.2	366	43.1	439	52.5	530	63.9	1846	43.1	
Female	630	74.9	560	67.8	485	56.9	412	47.5	321	36.1	2408	56.9	
Age (years)													<0.0001
65–74	388	45.0	456	49.8	493	58.3	545	65.1	583	68.7	2465	57.2	
75+	462	55.0	395	50.2	358	41.7	306	34.9	268	31.3	1789	42.8	
Marital status													0.5486
Married	841	99.0	845	99.2	845	99.1	847	99.7	847	99.4	4225	99.3	
Single	9	1.0	6	0.8	6	0.9	4	0.3	4	0.6	29	0.7	
Education level													<0.0001
Less than high-school graduate	623	77.4	604	76.0	593	73.0	540	63.4	499	58.3	2859	69.7	
High-school diploma	111	15.3	117	15.7	130	15.6	173	22.5	180	21.6	711	18.1	
College degree or higher	60	7.3	69	8.3	80	11.4	102	14.1	146	20.1	457	12.2	
Region													0.9898
City	614	77.0	606	76.3	615	77.3	608	76.5	618	76.0	3061	76.6	
Rural area	236	23.0	245	23.7	236	22.7	243	23.5	233	24.0	1193	23.4	
Employment status													<0.0001
Employed	188	24.4	241	28.2	260	29.3	292	33.4	344	40.7	1325	31.2	
Unemployed	606	75.6	549	71.8	544	70.7	523	66.6	482	59.3	2704	68.8	
Household income													<0.0001
Low	498	55.9	460	52.6	392	45.6	364	41.9	341	38.3	2055	47.0	
Middle–low	180	21.1	232	27.6	227	26.1	248	29.0	253	28.4	1140	26.4	
Middle–high	90	12.7	92	11.3	127	16.2	144	17.5	157	19.7	610	15.4	
High	74	10.2	64	8.5	98	12.1	90	11.6	99	13.6	425	11.2	

^(1)^ *p*-value by chi-square; ^(2)^ weighted %.

**Table 4:** Health-related behaviors of older Korean adults by quintile of plant protein intake.

Variables	Q1 (*n* = 850)		Q2 (*n* = 851)		Q3 (*n* = 851)		Q4 (*n* = 851)		Q5 (*n* = 851)		Total (*n* = 4254)		*p*-Value ^(1)^
	*n*	% ^(3)^	*n*	%	*n*	%	*n*	%	*n*	%	*n*	%	
Smoking													<0.0001
Current smoker	72	9.1	59	7	83	9.3	77	9.1	74	8.4	365	8.6	
Past smoker	151	17.5	202	23.7	231	26.9	287	34.4	347	41.8	1218	28.8	
Non-smoker	609	73.4	575	69.3	520	63.8	475	56.5	420	49.8	2599	62.7	
Drinking frequency													<0.0001
<1 time/month	616	74.6	600	71.9	530	63.5	523	61.7	469	53.2	2738	65.1	
1–4 times/month	112	13.2	114	13.5	161	18.7	171	21.3	198	24.3	756	18.2	
2–3 times/week	47	5.7	53	6.2	80	10.4	70	8.3	94	11.2	344	8.3	
≥4 times/week	58	6.5	70	8.4	64	7.5	75	8.7	81	11.3	348	8.5	
Stress level													0.0158
Severe stress	42	4.9	47	5.6	26	3.3	23	2.6	26	2.6	164	3.8	
Moderate stress	157	18.0	131	16.3	130	15.9	107	13.0	107	13.5	632	15.3	
Mild stress	366	45.3	402	46.5	409	49.1	430	53.0	445	51.4	2052	49.0	
No stress	265	31.8	255	31.6	267	31.7	277	31.4	262	32.6	1326	31.8	
Exercise													<0.0001
<1 day/week	731	92.3	682	85.4	661	80.0	631	76.9	625	74.9	3330	81.9	
1–2 day/week	13	1.4	25	3.4	27	3.5	31	4.2	40	4.8	136	3.5	
3–4 day/week	18	2.2	34	3.8	36	5.3	42	5.3	55	6.8	185	4.7	
≥5 day/week	34	4.1	51	7.4	83	11.3	110	13.6	106	13.6	384	10.0	
Weight status													0.094
Underweight (BMI < 18.5)	38	4.8	28	3.7	22	2.9	18	2.1	15	1.5	121	3.0	
Normal (18.5 ≤ BMI < 23)	282	32.8	283	32.2	289	34.3	298	35.4	282	34.4	1434	33.8	
Overweight (23 ≤ BMI < 25)	207	26.2	211	25.7	215	24.4	228	27.5	219	25.2	1080	25.8	
Obesity (BMI ≥ 25)	313	36.2	318	38.4	316	38.4	302	35.1	331	38.9	1580	37.4	
BMI	24.1 ± 0.1		24.1 ± 0.1		24.2 ± 0.1		24.1 ± 0.1		24.3 ± 0.1		24.2 ± 0.1		0.7589(+) ^(2)^
Dietary supplement use													0.0312
Yes	407	47.0	406	48.5	440	52.0	448	53.3	443	54.5	2144	51.0	
No	443	53.0	445	51.5	411	48.0	403	46.7	408	45.5	2110	49.0	
Chewing ability													0.0017
Very inconvenient	135	15.7	113	13.4	109	12.4	81	9.6	67	7.4	505	11.8	
Inconvenient	270	31.8	277	33.4	243	28.8	255	29.1	243	28.0	1288	30.3	
Normal	130	15.7	150	17.2	143	17.1	157	19.6	162	19.6	742	17.8	
Not inconvenient	128	15.0	132	16.1	154	18.1	158	18.7	162	19.9	734	17.5	
Not inconvenient at all	167	21.7	163	19.9	185	23.6	186	23.0	207	25.1	908	22.7	

^(1)^ *p*-value by chi-square; ^(2)^ *p* for trend was calculated by SURVEYREG procedure of SAS; ^(3)^ weighted %

**Table 5:** Measuring handgrip strength and assessing sarcopenia.

Variables	Q1 (*n* = 850)		Q2 (*n* = 851)		Q3 (*n* = 851)		Q4 (*n* = 851)		Q5 (*n* = 851)		Total (*n* = 4254)		*p*-Value ^(1)^
	*n*	% ^(4)^	*n*	%	*n*	%	*n*	%	*n*	%	*n*	%	
LHS ^(5)^	347	45.8	308	40.3	265	34.6	245	30.1	203	22.7	1368	34.7	<0.0001
NHS ^(6)^	418	54.2	483	59.7	532	65.4	573	69.9	613	77.3	2619	65.4	
Handgrip strength (Kg)	19.2 ± 0.3		20.4 ± 0.3		22.5 ± 0.4		24.4 ± 0.3		26.6 ± 0.4		22.6 ± 0.2		<0.0001(+) ^(2)^/0.0789(+) ^(2), (3)^

^(1)^ *p*-value by chi-square; ^(2)^ Unadjusted and adjusted *p* for trend were calculated by SURVEYREG procedure of SAS; ^(3)^ adjusted for gender, age and energy intake.; ^(4)^ weighted %; ^(5)^ LHS: low handgrip strength group, ^(6)^ NHS: normal- or higher handgrip strength group.

**Table 6:** Dietary behaviors of older Korean adults by quintile of plant protein intake.

Variables	Q1 (*n* = 850)		Q2 (*n* = 851)		Q3 (*n* = 851)		Q4 (*n* = 851)		Q5 (*n* = 851)		Total (*n* = 4254)		*p*-Value ^(1)^
	*n*	% ^(2)^	*n*	%	*n*	%	*n*	%	*n*	%	*n*	%	
Breakfast													<0.0001
5–7 times/week	748	87.0	795	91.4	809	95.0	813	95.2	809	95.1	3974	92.7	
3–4 times/week	39	4.7	21	2.9	20	2.4	14	1.8	23	2.7	117	2.9	
1–2 times/week	18	2.0	6	0.8	12	1.5	10	1.1	7	0.8	53	1.3	
Rarely (<1 time/week)	44	6.3	29	4.8	10	1.1	14	1.8	12	1.4	109	3.1	
Snack													<0.0001
Yes	719	84.2	753	88.5	764	90.7	782	92.1	801	93.8	3819	89.8	
No	131	15.8	98	11.5	87	9.3	69	7.9	50	6.2	435	10.2	
Home													<0.0001
Eating	724	83.4	782	91.5	779	92.1	809	95.0	811	95.7	3905	91.5	
Not eating	126	16.6	69	8.6	72	7.9	42	5.0	40	4.3	349	8.5	
Commercial location													<0.0001
Eating	549	65.2	562	66.5	600	71.9	611	72.3	654	76.5	2976	70.4	
Not eating	301	34.8	289	33.5	251	28.1	240	27.8	197	23.5	1278	29.6	
Institution location													0.2416
Eating	37	4.9	60	7.3	59	6.5	61	7.4	56	8.0	273	6.8	
Not eating	813	95.1	791	92.7	792	93.5	790	92.6	795	92.0	3981	93.2	
Eating-out frequency													<0.0001
≥1 time/day	20	2.5	36	4.4	50	6.7	40	5.1	57	6.8	203	5.1	
5–6 times/week	47	5.8	47	5.4	53	6.7	56	6.9	60	8.0	263	6.6	
3–4 times/week	45	5.7	56	6.6	73	7.7	74	9.2	68	8.3	316	7.5	
1–2 times/week	184	21.6	211	23.9	220	26.1	218	25.4	266	32.2	1099	25.8	
1–3 times/month	257	29.8	276	31.6	272	30.7	291	33.0	269	29.8	1365	30.9	
Rarely	296	34.6	225	28.1	183	22.1	172	20.5	131	14.9	1007	24.2	
Food security													<0.0001
Enough food secure	355	43.4	370	44.0	415	49.1	432	51.7	454	54.4	2026	48.4	
Mild food insecure	415	48.4	430	49.8	406	47.4	385	45.0	364	42.3	2000	46.6	
Moderate/severe food insecure	75	8.2	51	6.3	28	3.6	32	3.3	33	3.4	219	5.0	

^(1)^ *p*-value by chi-square; ^(2)^ weighted %.

**Table 7:** Protein intake by food group and its contribution by plant protein intake quintile.

Food Groups	Q1 (*n* = 850)		Q2 (*n* = 851)		Q3 (*n* = 851)		Q4 (*n* = 851)		Q5 (*n* = 851)		Total (*n* = 4254)		Unadjusted *p* for Trend ^(1)^	Adjusted *p* for Trend ^(1),(2)^
	Mean	SE	Mean	SE	Mean	SE	Mean	SE	Mean	SE	Mean	SE		
Total food (g)	740.8 ^d^	14.9	978.6 ^cd^	17.3	1177.9 ^c^	18.3	1413.0 ^b^	26.1	1844.1 ^a^	31.7	1223.8	15.0	<0.0001(+)	0.0006(+)
Cereals and grains (g)	155.0 ^d^	2.5	211.8 ^c^	2.7	256.9 ^c^	3.5	309.0 ^b^	3.6	418.5 ^a^	6.2	268.5	2.6	<0.0001(+)	<0.0001(+)
Potatoes and starches (g)	20.7 ^c^	2.3	29.2 ^b^	3.2	32.5 ^b^	3.3	36.4 ^b^	4.2	61.8 ^a^	5.6	35.9	1.9	<0.0001(+)	0.0017(+)
Sugars and sweets (g)	4.2 ^c^	0.4	6.0 ^bc^	0.6	6.3 ^bc^	0.4	7.8 ^b^	0.8	9.3 ^a^	0.7	6.7	0.3	<0.0001(+)	0.0006(−)
Legumes (g)	10.7 ^d^	1.3	23.2 ^c^	2.3	31.3 ^bc^	2.0	46.4 ^b^	2.8	100.9 ^a^	7.2	41.9	1.7	<0.0001(+)	0.0998(+)
Seeds and nuts (g)	2.6 ^d^	0.5	6.0 ^cd^	1.2	6.4 ^c^	0.9	8.2 ^b^	1.0	13.6 ^a^	1.5	7.3	0.5	<0.0001(+)	0.0998(+)
Vegetables (g)	155.6 ^d^	4.6	227.4 ^c^	5.9	292.3 ^bc^	7.3	357.7 ^b^	8.8	460.6 ^a^	10.5	296.8	4.3	<0.0001(+)	<0.0001(+)
Mushrooms (g)	1.8 ^d^	0.4	2.6 ^c^	0.4	3.7 ^bc^	0.6	4.5 ^b^	0.7	6.5 ^a^	1.1	3.8	0.3	<0.0001(+)	0.0079(+)
Fruits (g)	116.4 ^c^	6.9	150.0 ^bc^	9.1	184.1 ^b^	9.0	205.3 ^ab^	11.7	260.4 ^a^	14.2	182.3	6.0	<0.0001(+)	0.0482(+)
Seaweeds (g)	12.9 ^c^	2.1	25.1 ^bc^	3.4	28.7 ^bc^	3.6	38.9 ^b^	4.4	46.0 ^a^	5.6	30.1	2.1	<0.0001(+)	0.0002(+)
Meat and poultry (g)	49.2 ^c^	4.2	45.4 ^c^	3.9	54.0 ^b^	4.2	60.0 ^ab^	4.0	74.1 ^a^	5.9	56.4	2.1	<0.0001(+)	<0.0001(−)
Eggs (g)	11.9 ^c^	1.1	15.2 ^b^	1.1	16.6 ^b^	1.2	19.1 ^ab^	1.3	24.5 ^a^	1.7	17.4	0.7	<0.0001(+)	0.0052(−)
Fishes and shell fishes (g)	53.6 ^d^	4.8	74.0 ^c^	4.6	94.7 ^bc^	6.3	104.8 ^b^	6.6	128.0 ^a^	8.6	90.6	3.2	<0.0001(+)	0.0288(−)
Milks and dairy products (g)	55.4	4.5	56.4	4.6	51.9	4.6	55.9	4.8	59.1	5.5	55.7	2.3	0.5831(+)	<0.0001(−)
Oils and fats (g)	1.9 ^c^	0.1	2.8 ^bc^	0.2	3.3 ^b^	0.2	4.4 ^ab^	0.3	6.2 ^a^	0.3	3.7	0.1	<0.0001(+)	0.0430(−)
Beverages (g)	11.9 ^c^	0.5	18.5 ^bc^	0.6	24.4 ^b^	0.9	30.7 ^ab^	1.0	43.8 ^a^	1.5	25.7	0.5	<0.0001(+)	<0.0001(+)
Seasonings (g)	40.5 ^c^	4.5	57.2 ^bc^	4.9	57.5 ^bc^	4.7	68.3 ^b^	5.8	79.2 ^a^	7.3	60.3	2.6	<0.0001(+)	0.0030(−)
Other food (g)	0.4	0.2	0.3	0.1	0.2	0.1	0.4	0.1	0.2	0.1	0.3	0.1	0.6795(−)	0.1501(−)
Protein intake of food groups														
Total protein intake (g)	32.3 ^d^	0.7	42.7 ^cd^	0.8	51.0 ^c^	0.7	62.1 ^b^	0.9	84.9 ^a^	1.1	54.3	0.6	<0.0001(+)	<0.0001(+)
Plant protein (g)	16.2	0.2	24.1	0.1	30.5	0.1	38.8	0.1	57.7	0.6	33.2	0.3	<0.0001(+)	<0.0001(+)
Animal protein (g)	14.7	0.6	17.4	0.7	19.2	0.7	21.7	0.8	26.1	1.0	19.7	0.4	<0.0001(+)	<0.0001(+)
Other protein (g)	1.4	0.2	1.3	0.2	1.2	0.2	1.6	0.2	1.1	0.2	1.3	0.1	<0.3671(−)	<0.1451(−)
Cereals and grains (g)	10.1 ^c^	0.2	14.1 ^b^	0.2	17.0 ^b^	0.2	21.0 ^ab^	0.2	29.3 ^a^	0.4	18.2	0.2	<0.0001(+)	<0.0001(+)
Potatoes and starches (g)	0.3 ^c^	0.03	0.5 ^b^	0.1	0.5 ^b^	0.1	0.6 ^b^	0.1	1.0 ^a^	0.1	0.6	0.03	<0.0001(+)	0.1142(+)
Sugars and sweets (g)	0.01	0.004	0.02	0.004	0.04	0.01	0.1	0.01	0.1	0.02	0.04	0.01	0.0122(+)	0.4843(+)
Legumes (g)	1.1 ^d^	0.07	2.3 ^c^	0.11	3.5 ^bc^	0.1	5.6 ^b^	0.2	11.1 ^a^	0.5	4.7	0.1	<0.0001(+)	<0.0001(+)
Seeds and nuts (g)	0.3 ^d^	0.03	0.6 ^c^	0.1	0.8 ^c^	0.1	1.0 ^b^	0.1	2.1 ^a^	0.2	0.9	0.1	<0.0001(+)	0.0001(+)
Vegetables (g)	2.3 ^d^	0.1	3.4 ^c^	0.1	4.5 ^bc^	0.1	5.5 ^b^	0.1	7.3 ^a^	0.2	4.6	0.1	<0.0001(+)	<0.0001(+)
Mushrooms (g)	0.1	0.01	0.1	0.01	0.1	0.02	0.1	0.02	0.2	0.03	0.1	0.01	<0.0001(+)	0.0034(+)
Fruits (g)	0.6 ^b^	0.04	0.8 ^b^	0.1	1.0 ^ab^	0.1	1.1 ^ab^	0.1	1.6 ^a^	0.1	1.0	0.04	<0.0001(+)	0.1714(+)
Seaweeds (g)	0.3	0.02	0.5	0.04	0.6	0.1	0.8	0.1	0.8	0.1	0.6	0.02	<0.0001(+)	<0.0001(+)
Meat and poultry (g)	7.3 ^c^	0.5	7.3 ^c^	0.5	8.1 ^bc^	0.5	9.4 ^b^	0.6	11.1 ^a^	0.7	8.6	0.3	<0.0001(+)	<0.0001(−)
Eggs (g)	1.6 ^c^	0.2	2.1 ^bc^	0.2	2.3 ^b^	0.2	2.6 ^b^	0.2	3.3 ^a^	0.2	2.4	0.1	<0.0001(+)	0.0049(−)
Fishes and shell fishes (g)	4.0 ^c^	0.3	6.2 ^bc^	0.4	7.1 ^b^	0.5	7.9 ^b^	0.4	9.7 ^a^	0.7	6.9	0.2	<0.0001(+)	<0.0001(−)
Milks and dairy products (g)	1.7	0.1	1.8	0.2	1.7	0.2	1.8	0.2	2.0	0.2	1.8	0.1	0.3490(+)	<0.0001(−)
Oils and fats (g)	0.002	0.001	0.01	0.002	0.01	0.004	0.01	0.004	0.01	0.004	0.01	0.001	0.5777(+)	0.6722(+)
Beverages (g)	0.9 ^c^	0.04	1.5 ^c^	0.1	2.0 ^bc^	0.1	2.5 ^b^	0.1	3.7 ^a^	0.1	2.1	0.1	<0.0001(+)	<0.0001(+)
Seasonings (g)	0.3	0.02	0.4	0.02	0.4	0.02	0.4	0.02	0.5	0.03	0.4	0.01	<0.0001(+)	<0.0001(−)
Other food (g)	0.04	0.03	0.01	0.003	0.01	0.003	0.02	0.01	0.01	0.004	0.02	0.01	0.4127(−)	0.2464(−)
Percentage contribution of food groups to total protein intake														
Cereals and grains (%)	31.2	0.9	33.0	0.8	33.3	0.8	33.9	0.6	34.5	0.6	33.5	0.4	0.2647(−)	<0.0001(+)
Potatoes and starches (%)	0.9	0.2	1.1	0.2	1.0	0.1	0.9	0.2	1.1	0.1	1.0	0.1	0.7111(−)	0.0536(−)
Sugars and sweets (%)	0.03	0.01	0.05	0.01	0.1	0.01	0.1	0.02	0.1	0.02	0.1	0.01	0.2524(+)	0.3165(−)
Legumes (%)	3.5 ^e^	0.3	5.3 ^d^	0.4	6.9 ^c^	0.4	9.0 ^b^	0.4	13.1 ^a^	0.6	8.6	0.2	<0.0001(+)	<0.0001(+)
Seeds and nuts (%)	0.8 ^c^	0.1	1.3 ^b^	0.1	1.5 ^b^	0.2	1.6 ^b^	0.1	2.4 ^a^	0.2	1.7	0.1	<0.0001(+)	<0.0001(+)
Vegetables (%)	7.0	0.3	8.0	0.2	8.8	0.3	8.8	0.2	8.6	0.2	8.4	0.1	0.0230(+)	<0.0001(+)
Mushrooms (%)	0.2	0.03	0.2	0.03	0.2	0.04	0.2	0.03	0.2	0.04	0.2	0.02	0.0487(+)	0.0076(+)
Fruits (%)	1.9	0.1	1.8	0.2	2.0	0.1	1.8	0.1	1.8	0.2	1.9	0.1	0.3953(−)	0.9750(−)
Seaweed (%)	0.9	0.1	1.1	0.1	1.2	0.1	1.3	0.1	1.0	0.1	1.1	0.1	0.2673(+)	<0.0531(+)
Meat and poultry (%)	22.7 ^a^	0.9	17.0 ^b^	0.7	16.0 ^b^	0.7	15.2 ^b^	0.6	13.1 ^c^	0.6	15.9	0.3	<0.0001(−)	<0.0001(−)
Eggs (%)	5.0	0.4	4.8	0.3	4.5	0.3	4.2	0.3	3.9	0.3	4.4	0.2	0.0870(−)	<0.0001(−)
Fishes and shell fishes (%)	12.4	0.6	14.4	0.6	14.0	0.6	12.7	0.5	11.4	0.6	12.8	0.3	0.3299(−)	<0.0001(−)
Milks and dairy products (%)	5.3 ^a^	0.4	4.3 ^b^	0.3	3.3 ^bc^	0.3	2.9 ^bc^	0.3	2.3 ^c^	0.2	3.3	0.1	<0.0001(−)	<0.0001(−)
Oils and fats (%)	0.01	0.004	0.02	0.004	0.02	0.01	0.02	0.01	0.01	0.004	0.02	0.004	0.5239(−)	0.8015(+)
Beverages (%)	2.8 ^c^	0.2	3.5 ^bc^	0.2	3.9 ^b^	0.2	4.1 ^b^	0.2	4.4 ^a^	0.2	3.9	0.1	<0.0001(+)	<0.0001(+)
Seasonings (%)	1.0	0.1	1.0	0.1	0.8	0.1	0.7	0.04	0.6	0.04	0.8	0.03	<0.0001(−)	<0.0001(−)
Other food (%)	0.1	0.1	0.02	0.01	0.02	0.01	0.03	0.01	0.01	0.01	0.04	0.01	0.2033(−)	0.2345(−)

^(1)^ Unadjusted and adjusted *p* for trend were calculated by SURVEYREG procedure of SAS; ^(2)^ total food intake and other variables were adjusted for gender, age and energy intake. ^a–e^ different superscript letters mean significantly different among groups at the *p* < 0.05 level by Tukey’s multiple range comparison.

**Table 8:** Nutrient intake of older Korean adults by quintile of plant protein.

Nutrients	Q1 (*n* = 850)		Q2 (*n* = 851)		Q3 (*n* = 851)		Q4 (*n* = 851)		Q5 (*n* = 851)		Total (*n* = 4254)		Unadjusted *p* for Trend ^(1)^	Adjusted *p* for Trend ^(1),(2)^
	Mean	SE	Mean	SE	Mean	SE	Mean	SE	Mean	SE	Mean	SE		
Energy (kcal)	1028.9 ^d^	14.2	1332.9 ^c^	14.5	1579.8 ^bc^	14.7	1865.6 ^b^	18.7	2473.3 ^a^	25.8	1647.7	14.4	<0.0001(+)	<0.0001(+)
Carbohydrate (g)	176.4 ^d^	2.1	233.3 ^c^	2.2	277.4 ^c^	2.4	325.2 ^b^	3.1	427.9 ^a^	4.4	286.6	2.5	<0.0001(+)	<0.0001(+)
Protein (g)	32.3 ^d^	0.7	42.7 ^cd^	0.8	51.0 ^cd^	0.7	62.1 ^b^	0.9	84.9 ^a^	1.1	54.3	0.6	<0.0001(+)	<0.0001(+)
Fat (g)	16.6 ^c^	0.7	21.1 ^bc^	0.8	24.3 ^b^	0.7	29.6 ^b^	0.8	41.1 ^a^	1.1	26.4	0.4	<0.0001(+)	<0.0001(+)
Fiber (g)	13.5 ^c^	0.4	19.2 ^bc^	0.5	23.8 ^bc^	0.4	29.1 ^b^	0.4	41.3 ^a^	0.8	25.2	0.4	<0.0001(+)	<0.0001(+)
Calcium (mg)	263.6 ^c^	6.8	353.8 ^bc^	8.4	425.7 ^b^	10.3	499.9 ^b^	14.2	658.7 ^a^	14.4	438.1	6.5	<0.0001(+)	<0.0001(+)
Phosphorus (mg)	518.8 ^c^	9.0	699.8 ^bc^	10.1	837.2 ^b^	10.6	1017.4 ^ab^	12.5	1394.0 ^a^	16.9	888.3	9.4	<0.0001(+)	<0.0001(+)
Iron (mg)	6.3 ^c^	0.2	8.3 ^bc^	0.2	10.6 ^b^	0.2	12.7 ^ab^	0.2	17.1 ^a^	0.3	10.9	0.2	<0.0001(+)	<0.0001(+)
Sodium (mg)	1564.8 ^d^	38.2	2116.5 ^cd^	45.8	2703.0 ^c^	72.7	3257.6 ^b^	71.7	4337.2 ^a^	84.6	2779.9	36.1	<0.0001(+)	<0.0001(+)
Magnesium (mg)	160.5 ^d^	2.3	230.5 ^c^	2.9	283.5 ^bc^	3.2	352.4 ^b^	4.1	492.1 ^a^	6.3	301.9	3.3	<0.0001(+)	<0.0001(+)
Potassium (mg)	1447.9 ^c^	28.1	2015.6 ^bc^	34.3	2452.2 ^b^	36.5	2942.5 ^ab^	46.9	3975.8 ^a^	58.2	2552.2	32.2	<0.0001(+)	<0.0001(+)
Zinc (mg)	5.6 ^c^	0.1	7.4 ^bc^	0.1	9.0 ^b^	0.1	10.7 ^ab^	0.1	13.9 ^a^	0.2	9.3	0.1	<0.0001(+)	<0.0001(+)
Vitamin A (μg RAE)	161.3 ^d^	6.1	233.4 ^c^	7.9	286.6 ^bc^	9.4	336.6 ^b^	11.5	446.9 ^a^	21.2	291.3	6.6	<0.0001(+)	0.8488(−)
Carotene (μg)	1245.8 ^c^	51.5	1942.2 ^bc^	71.9	2590.1 ^b^	95.9	3059.6 ^ab^	111.9	3968.2 ^a^	143.5	2546.1	54.6	<0.0001(+)	0.2332(−)
Retinol (μg)	57.5 ^c^	3.8	71.4 ^bc^	5.0	70.6 ^bc^	4.6	81.4 ^b^	5.7	116.2 ^a^	18.1	79.1	4.3	0.0036(+)	<0.0001(−)
Thiamine (mg)	0.7	0.01	0.9	0.01	1.1	0.02	1.3	0.02	1.7	0.03	1.1	0.01	<0.0001(+)	<0.0001(+)
Riboflavin (mg)	0.7 ^c^	0.02	0.9 ^b^	0.02	1.1 ^ab^	0.02	1.3 ^ab^	0.03	1.8 ^a^	0.03	1.2	0.02	<0.0001(+)	0.0415(+)
Niacin (mg)	6.3 ^c^	0.1	8.4 ^b^	0.2	9.8 ^b^	0.2	11.8 ^ab^	0.2	15.2 ^a^	0.3	10.3	0.1	<0.0001(+)	0.9370(+)
Vitamin D (μg)	1.5 ^c^	0.1	2.3 ^b^	0.2	2.4 ^b^	0.2	2.6 ^b^	0.2	3.7 ^a^	0.4	2.5	0.1	<0.0001(+)	0.0004(−)
Vitamin E (mg α-TE)	2.6 ^e^	0.1	3.9 ^d^	0.1	4.7 ^c^	0.1	5.7 ^b^	0.1	7.9 ^a^	0.2	4.9	0.1	<0.0001(+)	0.0002(+)
Vitamin C (mg)	30.0 ^e^	1.5	43.0 ^d^	1.9	52.2 ^c^	2.0	65.8 ^b^	2.9	80.8 ^a^	3.2	54.1	1.3	<0.0001(+)	0.0738(+)
Folic acid (μg DFE)	160.0 ^d^	3.4	226.2 ^c^	3.8	286.8 ^bc^	4.2	357.2 ^b^	5.2	492.4 ^a^	7.3	302.6	3.8	<0.0001(+)	<0.0001(+)
Energy distribution (%)														
Carbohydrate	74.2	0.5	73.7	0.5	73.7	0.4	72.7	0.4	71.5	0.3	73.2	0.2	<0.0001(+)	<0.0001(+)
Protein	12.4	0.2	12.7	0.2	13.0	0.1	13.4	0.1	13.8	0.1	13.1	0.1	<0.0001(+)	<0.0001(+)
Plant protein	6.8	0.	7.6	0.1	8.1	0.1	8.8	0.1	9.7	0.1	8.2	0.1	<0.001(+)	<0.001(+)
Animal protein	5.2	0.2	4.8	0.2	4.6	0.2	4.4	0.1	4.0	0.1	4.6	0.1	0.0581(+)	0.0431(+)
Other protein ^(3)^	0.4	0.1	0.3	0.1	0.3	0.04	0.3	0.04	0.2	0.02	0.3	0.03	0.9592(+)	0.8474(+)
Fat	13.4	0.4	13.6	0.4	13.3	0.3	13.9	0.3	14.7	0.3	13.8	0.2	0.0086(+)	<0.0001(+)

^(1)^ Unadjusted and adjusted *p* for trend were calculated by SURVEYREG procedure of SAS; ^(2)^ energy intake was adjusted for gender, age, and total food intake; in addition, other variables were adjusted for gender, age, and energy intake; ^(3)^ other protein included miscellaneous foods, baked goods, and alcoholic beverages; ^a–e^ different superscript letters mean significantly different among groups at the *p* < 0.05 level by Tukey’s multiple range comparison.

**Table 9:** Energy contribution of protein older Korean adults by quintile of plant protein.

	Q1 (*n* = 850)		Q2 (*n* = 851)		Q3 (*n* = 851)		Q4 (*n* = 851)		Q5 (*n* = 851)		Total (*n* = 4254)	
	*n*	% ^(2)^	*n*	%	*n*	%	*n*	%	*n*	%	*n*	%
<7%	30	3.2	13	2.1	8	0.8	4	0.5	3	0.3	58	1.4
7~20% ^(1)^	773	90.9	806	93.8	810	95.1	816	95.6	815	95.2	4020	94.1
>20%	47	5.8	32	4.1	33	4.2	31	3.9	33	4.5	176	4.5

^(1)^ The Acceptable Macronutrient Distribution Range (AMDR) of protein in Korea is 7–20%; ^(2)^ weighted %.

**Table 10:** KHEI total and component scores of older Korean adults by quintile of plant protein intake.

	Q1 (*n* = 850)		Q2 (*n* = 851)		Q3 (*n* = 851)		Q4 (*n* = 851)		Q5 (*n* = 851)		Total (*n* = 4254)		Unadjusted *p* for Trend ^(1)^	Adjusted *p* for Trend ^(1),(2)^
	Mean	SE	Mean	SE	Mean	SE	Mean	SE	Mean	SE	Mean	SE		
Total score	60.0 ^b^	0.5	66.2 ^ab^	0.5	69.5 ^a^	0.5	70.9 ^a^	0.4	70.7 ^a^	0.5	67.4	0.3	<0.0001(+)	<0.0001(+)
Have breakfast	9.0	0.1	9.3	0.1	9.7	0.1	9.7	0.1	9.7	0.1	9.5	0.04	<0.0001(+)	0.0031(+)
Mixed grains intake	1.7 ^c^	0.1	2.4 ^b^	0.1	2.7 ^b^	0.1	3.0 ^ab^	0.1	3.3 ^a^	0.1	2.6	0.1	<0.0001(+)	<0.0001(+)
Total fruits intake	2.4	0.1	2.6	0.1	2.8	0.1	2.8	0.1	3.1	0.1	2.8	0.1	<0.0001(+)	0.1695(−)
Fresh fruits intake	2.5	0.1	2.7	0.1	2.9	0.1	2.9	0.1	3.1	0.1	2.8	0.1	<0.0001(+)	0.3822(−)
Total vegetables intake	2.5 ^c^	0.1	3.3 ^b^	0.1	3.8 ^ab^	0.1	4.1 ^ab^	0.1	4.4 ^a^	0.04	3.6	0.03	<0.0001(+)	<0.0001(+)
Unsalted vegetables intake ^(3)^	2.2 ^c^	0.1	3.1 ^b^	0.1	3.4 ^b^	0.1	3.8 ^ab^	0.1	4.0 ^a^	0.1	3.3	0.04	<0.0001(+)	<0.0001(+)
Meat, fish, eggs, and beans intake	4.7 ^d^	0.2	5.8 ^c^	0.2	6.6 ^bc^	0.1	7.4 ^b^	0.1	8.4 ^a^	0.1	6.6	0.1	<0.0001(+)	<0.0001(+)
Milk and milk products intake	2.6	0.2	2.7	0.2	2.5	0.2	2.6	0.2	2.5	0.2	2.6	0.1	0.7462(−)	<0.0001(−)
Percentage of energy from saturated fatty acid	8.4	0.1	8.9	0.1	9.3	0.1	9.3	0.1	9.4	0.1	9.1	0.1	<0.0001(+)	<0.0001(+)
Sodium intake	9.6 ^a^	0.1	9.0 ^a^	0.1	8.1 ^b^	0.1	7.2 ^bc^	0.1	5.3 ^c^	0.1	7.9	0.1	<0.0001(−)	<0.0001(−)
Percentage of energy from sweets and beverages	9.2	0.1	9.3	0.1	9.6	0.1	9.6	0.1	9.8	0.04	9.5	0.03	<0.0001(+)	<0.0001(+)
Percentage of energy from carbohydrate	1.5	0.1	1.5	0.1	1.6	0.1	1.8	0.1	2.0	0.1	1.7	0.04	<0.0001(+)	0.0268(−)
Percentage of energy from fat	2.2	0.1	2.3	0.1	2.4	0.1	2.6	0.1	2.9	0.1	2.5	0.1	<0.0001(+)	0.0388(−)
Energy intake	1.6 ^c^	0.1	3.3 ^b^	0.1	4.2 ^a^	0.1	4.1 ^a^	0.1	2.7 ^bc^	0.1	3.2	0.04	<0.0001(+)	<0.0001(+)

^(1)^ Unadjusted and adjusted *p* for trend were calculated by SURVEYREG procedure of SAS; ^(2)^ adjusted for gender, age, and energy intake; ^(3)^ Unsalted Vegetables intake excluding Kimchi and pickled vegetables intake; ^a–d^ different superscript letters mean significantly different among groups at the *p* < 0.05 level by Tukey’s multiple range comparison.

## Data Availability

All data were obtained from the Korea Disease Control and Prevention Agency and are available with the permission of the Korea Disease Control and Prevention Agency. The data in this study were from the Korea National Health and Nutrition Examination Survey.
